# Investigation of Occult Hip Fractures: The Use of CT and MRI

**DOI:** 10.1155/2013/830319

**Published:** 2013-02-07

**Authors:** S. K. Gill, J. Smith, R. Fox, T. J. S. Chesser

**Affiliations:** Trauma and Orthopaedic Department, Frenchay Hospital, North Bristol NHS Trust, Frenchay Park Road, Bristol BS16 1LE, UK

## Abstract

*Aim*. At present there is no data looking at modern multislice computerised tomography (CT) in the investigation of occult hip fracture. The aim of this study was to retrospectively compare the reports of patients sent for magnetic resonance imaging (MRI) or CT with negative radiographs and a clinical suspicion of a fractured neck of femur. *Methods*. All patients presenting to the hospital with a clinical suspicion of a hip fracture but initial negative radiographs over a three-year period were included. Patients were either investigated with an MRI scan or CT scan. The presence of a fracture, the requirement for surgery, and any further requirement for imaging were recorded. *Results*. Over three years 92 patients were included of which 61 were referred for a CT and 31 for an MRI. Thirty-four patients were found to have a fracture. Of these, MRI picked up a fracture in 36% and CT in 38% of referrals. *Discussion*. Up to 10% of proximal femur fractures may be missed on initial radiographs. Current guidelines state patients should be offered MRI if hip fracture is suspected despite negative hip radiographs. Our findings show that modern multislice CT may be comparable with MRI for detecting occult fracture.

## 1. Introduction

The early diagnosis and treatment of patients sustaining fractures of the proximal femur (hip fractures) has led to improved results both in terms of morbidity and mortality. Early treatment helps with pain control and well as reducing the length of hospital stay. However, it is recognized 2–10% of fractures may not be clearly visible on initial radiographs, and further imaging is required to make a definitive diagnosis [1–6]. These fractures have been termed occult hip fractures.

Current guidelines state patients should be offered magnetic resonance imaging (MRI) if hip fracture is suspected despite negative anteroposterior pelvis and lateral hip radiographs [1]. However, MRI may not be accessible in hospitals out of office hours, is more expensive than other imaging, and is contraindicated in some patients. One alternative is multislice computerised tomography (CT) which is more readily available, though there is no evidence of its sensitivity. 

The aim of this study was to look at the sensitivity and results of suspected occult hip fractures (with negative radiographs) imaged with either MRI or CT. 

## 2. Materials and Methods

A retrospective study of all patients presenting to a single hospital, over a three-year period with a clinical suspicion of a hip fracture, was performed. Patients presenting with clinical suspicion of a hip fracture (persistent hip pain after trauma, inability to bear weight, and pain on attempted straight leg raising, passive rotation, or axial loading tests), initially underwent both anteroposterior pelvis and lateral hip radiographs. If these were negative or inconclusive, further imaging was then arranged. All patients were subsequently sent for either MRI or CT scanning depending on accessibility. CT scans were used in those patients where MRI was contraindicated or where there was going to be more than a 24-hour wait. 

The CT examinations were conducted by one of two multislice helical Siemens scanners with a ×4 quad slice and ×1 62 slice. The MRI examination was conducted using T1 weighted spine echo + STIR (Short Tau Inversion Recovery) axial and coronal scan, using a Philips 3T or a Philips 1.5T. 

## 3. Results

The hospital treated 1353 hip fractures over the three-year period. During this time 92 patients were referred for further imaging for a diagnosis of an occult hip fracture of which 34 (37%) identified a fracture. This gave an occult fracture rate of 2.5%, with 7% of those presenting with a clinical suspicion of a hip fracture requiring further investigation after initial negative radiographs. When the radiographs were retrospectively reviewed by experienced musculoskeletal radiologists or trauma orthopaedic surgeons, 11 of the fractures were evident on the initial films, dropping the actual occult fracture rate to 1.7%.

CT scans were performed in 61 and MRI in 31 patients. Of those undergoing CT, 23 (38%) were diagnosed with a fracture compared with 11 who underwent MRI (36%). Of those who had a positive diagnosis 28 out of 34 underwent operative treatment with 17 sustaining intracapsular and 11 extracapsular injuries (see Table 1).

All patients who had scans for possible fractures were reviewed on the system to see if they had any further followup radiographs of their hips in the six weeks following their scans. Four patients who had a negative CT had further radiographs of their hip, which in all cases was negative. 

Of the six people who were not operated on, one had an isolated greater trochanter fracture, two had scans which were suspicious of fracture, but the patients were mobilising comfortably, two had intracapsular fractures which were over three weeks old, the patients were mobilising comfortably, and one patient had an intertrochanteric fracture which was not operated on due to the patient having multiple medical problems and being bed bound due to end stage cancer. 

## 4. Discussion

About 70–75,000 people fracture their proximal femur annually in the UK [1], and this number is expected to rise [2, 3]. It is quoted that up to 10% of these may be missed on initial radiographs and require further imaging if there is clinical suspicion of a hip fracture [2–6].

Occult fractures which are initially missed have implications for patients and clinicians. Delays in diagnosis can lead to AVN, nonunion and a greater risk of arthroplasty, unnecessary pain, increased mortality, and thromboembolic complications [2, 7–9]. The primary goal of fracture management is to return patients to prefracture level of function. Patients with a proximal femur fracture and a delay of greater than 48 hours from hospital admission to surgery have doubled the risk of dying before the end of the first postop year [10]. Early diagnosis of occult hip fractures would shorten hospitalisation by expediting definitive treatment, leading to reduced costs [6]. Kim et al., in a study looking at initially missed occult hip fractures, showed that there was an increased risk of need for operation in patients who were diagnosed on a subsequent occasion when they returned after discharge [4]. Patients who do not have a hip fracture do not necessarily need to be kept immobile or require a stay in hospital.

MRI is used to diagnose a multitude of all occult fractures, and the majority of papers reviewed on this subject felt that MRI was the most useful adjunct in the diagnosis of occult proximal femur fractures [2, 3, 11–21]. However, MRIs may not be readily available in all hospitals, is expensive, and is contraindicated in confused patients and patients with cardiac pacemakers [10, 12].

Our criteria for further imaging with MRI or CT were the same as previously published by Chana et al. [10]: negative plain radiographs (anteroposterior pelvic and lateral hip views) and a high clinical suspicion of fracture (persistent hip pain after trauma, inability to bear weight, and pain on attempted straight leg raising, passive rotation, or axial loading tests). Such patients underwent an urgent MRI or CT scan, within 48 hours where possible. The demographics of our two groups were similar, the indication for further imaging was the same, and the modality for which they were scanned was due solely to the availability of the MRI scanner or if there were any contraindications for using MRI. Our results showed that the proportion of scans in each modality that showed a fracture was similar (38% of CT scans, 36% of MRI scans). No MRI or CT scan missed an occult fracture which was then found when the initial X-rays were reported. No patients who underwent an MRI scan had their scans repeated or had further radiographs in the six weeks following their scans. Four patients who had a negative CT had a further radiograph of their hip with six weeks of their scans; these were all reported negative for a fracture. 

Lubovsky et al. compared CT to MRI in the early diagnosis of occult hip fractures [6]. They scanned six patients with a possible fractured proximal femur with CT and then immediately after with MRI. Their findings showed that the CT images resulted in misdiagnosis in 66% of patients. Out of the four greater trochanteric fractures diagnosed by CT, three were rediagnosed as pertrochanteric and one as subcapital fractures after MRI. Lubovsky et al. felt that an MRI was more cost effective, as it gave a definitive diagnosis, including identifying soft tissue injuries, by one short examination [6]. However, their group for comparison was very small (only 13 patients in total, only six patients scanned by CT).

Cabarrus et al. compared CT with MRI for insufficiency fractures in the pelvis and reported 64 cases where MRI and CT could be compared side by side [7]. MRI identified 128/129 patients with pelvic fractures, CT identified 89/129. Only 2% of patients had fractures detected on CT but not on MRI, 52% had fractures detected on both, and 47% had fractures on MRI but not CT. CT only detected 70% of femoral neck fractures (90% on MRI) and 42.9% of femoral head fractures (100% on MRI). But the time lapse of up to 3 months between CT and MRI in some patients may have affected the results. Cabarrus et al. found that MRI did miss a fracture due to partial volume effects and adjacent joint effusion along with motion artefacts. This fracture was picked up by CT. 

CT scanning was felt to be less useful than MRI by Pandey et al. due to the CT scanning only being available in the axial plane which may miss small impacted fractures or undisplaced fractures than run parallel to the axial plane [16]. However, this paper was written in 1995 before, the widespread usage of multislice helical scanners were available. Our results show that neither CT nor MRI missed a proximal hip fracture. 

The majority of the papers in this field are over 10 years old [8, 9, 16, 19–27]. Advances in technology, such as 64-slice scanners and sophisticated 3-dimensional reconstruction algorithms, may have made the reliability of CT comparable to that of MRI. Perron et al. in 2005 thought CT was sensitive in depicting fracture lines and in the assessment of biomechanical stability [28]. Memarsadeghi et al. [29] in 2006 compared multidetector CT with MRI in detecting occult scaphoid fractures. In their study they demonstrated that multidetector CT was better than MRI for visualising the difference between a purely trabecular and cortical fracture, although MRI had better sensitivity overall for the visualisation of fractures [29]. They concluded that CT was useful diagnostically due to it being cheaper and more readily available. This finding was supported by a more recent paper by Mallee et al., who found that CT had comparable accuracy to MRI in diagnosing occult scaphoid fractures [30]. The cost for a CT scan (one area, no contrast) is *£*101. The cost of an MRI (one area, no contrast) is *£*206 [31]. If CT can be shown to be as reliable and accurate as MRI, this has considerable implications because of its widespread availability out of hours and lower cost. 

Other imaging modalities previously described include bone scanning where it is reported high sensitivity at 72 hours; however, other studies have shown a false negative rate at 24 hours suggesting it is not as sensitive as MRI for an early diagnosis [11, 32]. 

As this is a retrospective study, we were unable to ensure every patient who had a negative CT or MRI scan had further imaging to rule out a fracture. However, our rate of 37% of patients with negative initial radiographs having a fracture on further imaging is comparable with results reported by other studies (range from 13% to 67%) [6–8, 11, 13, 16, 18, 20, 21, 23, 33]. We were unable to determine retrospectively what proportion of patients had a suspected fracture but did not undergo an MRI scan. However, it has been the policy at our institution to perform an MRI or a CT scan on all patients with a suspected fracture of the femoral neck but with normal plain radiographs, so we believe that this proportion would be negligible. Due to the retrospective nature of our study we were unable to perform both CT and MRI on all of our patients, so we are unable to directly compare the sensitivity of both modalities. 

## 5. Conclusion

This is the first study reporting the use of multislice CT scanning and suggests it has a role to play in the investigation of occult hip fractures. CT scanning has widespread availability out of hours and lower cost when compared to MRI scanning. It can also be used in patients with confusion and cardiac pacemakers, where MRI is contraindicated. Whilst our study population was too small for statistical confirmation our findings show that CT may be considered as a useful adjunct. We feel that further prospective studies comparing CT and MRI for diagnosing occult fractures are needed to define their roles further. 

## Figures and Tables

**Table 1:** Results.

	Total	CT	MRI
Number	92	61	31
Male	33	23	10
Female	59	38	21
Age range	52–100	52–100	61–95
Mean age	82	83	81
Fracture found	34 (37%)	23 (38%)	11 (36%)
Operated on	28	19	9
Intracapsular	17	14	6
Extracapsular	11	5	3
